# Supplementary material for: The interactome of intact mitochondria by cross-linking mass spectrometry provides evidence for coexisting respiratory supercomplexes
Source: Mol Cell Proteomics. 2017 Dec 8;17(2):216–32. doi: 10.1074/mcp.RA117.000470 (PMC5795388; doi:10.1074/mcp.RA117.000470)
Supplement: Supplemental Data [file supp_RA117.000470_133922_0_supp_23978_fzffwf.zip › spectra_annotation/mito_spectra_annotation/78-1-1-1-6-1.pdf]

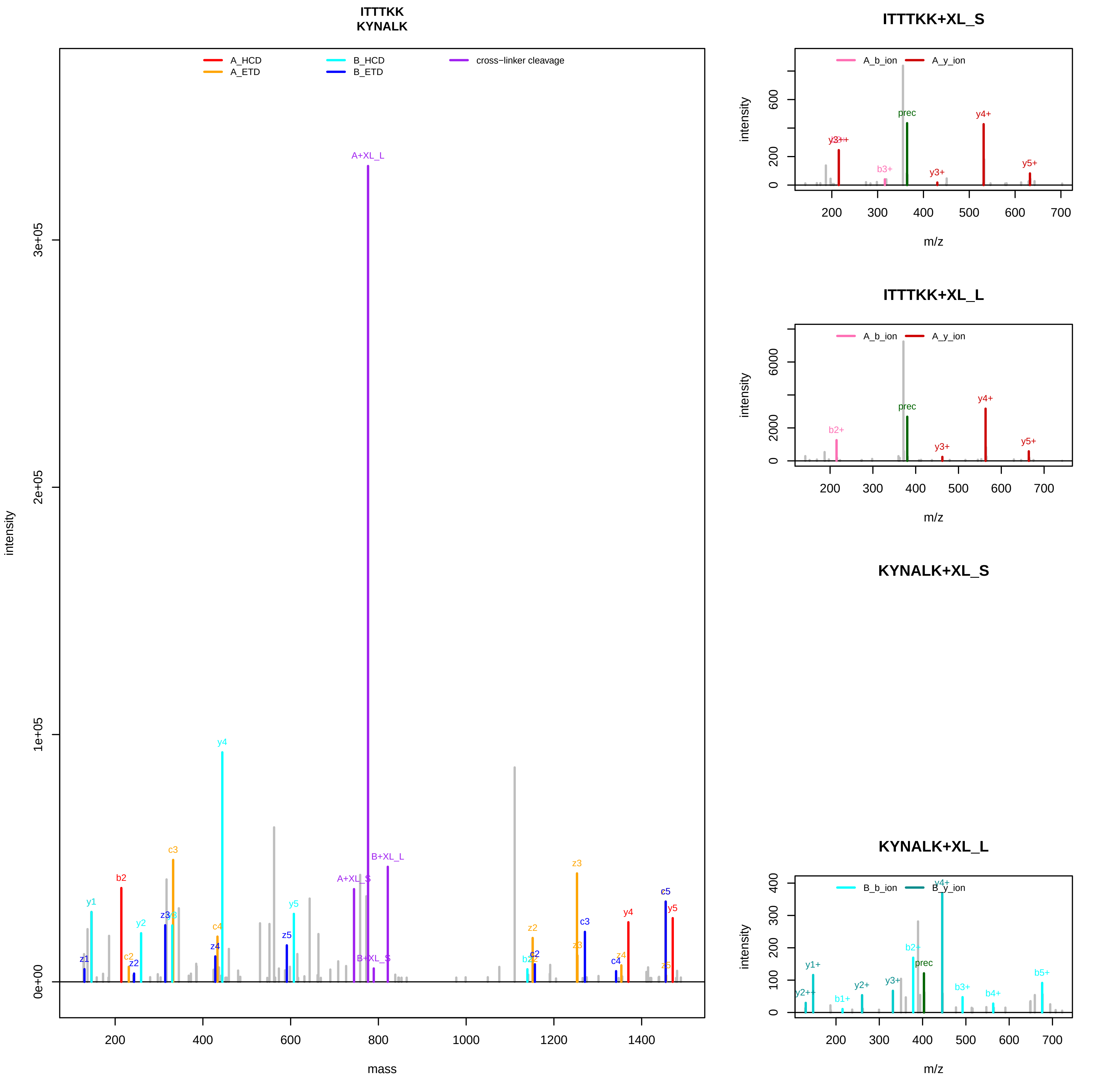

ITTTKK
KYNALK
A_HCD
A_ETD
B_HCD
B_ETD
cross−linker cleavage
A+XL_L
A+XL_S
B+XL_L
B+XL_S
y1
y2
y4
y5
b2
c2
c3
c4
c5
z1
z2
z3
z4
z5
intensity
mass
0e+00
1e+05
2e+05
3e+05
200
400
600
800
1000
1200
1400
ITTTKK+XL_S
A_b_ion
A_y_ion
prec
y3++
y3+
b3+
y4+
y5+
ITTTKK+XL_L
b2+
KYNALK+XL_S
KYNALK+XL_L
B_b_ion
B_y_ion
y1+
y2++
y2+
b1+
y3+
b2+
y4+
b3+
b4+
b5+
m/z
